# (*Z*)-2-(5-Chloro-2-oxoindolin-3-yl­idene)-*N*-methyl­hydrazinecarbothio­amide

**DOI:** 10.1107/S1600536812007386

**Published:** 2012-03-07

**Authors:** Amna Qasem Ali, Naser Eltaher Eltayeb, Siang Guan Teoh, Abdussalam Salhin, Hoong-Kun Fun

**Affiliations:** a School of Chemical Sciences, Universiti Sains Malaysia, Minden, Penang, Malaysia; b Faculty of Science, Sabha University, Libya; c Department of Chemistry, International University of Africa, Khartoum, Sudan; d X-ray Crystallography Unit, School of Physics, Universiti Sains Malaysia, 11800 USM, Penang, Malaysia

## Abstract

In the title compound, C_10_H_9_ClN_4_OS, an intra­molecular N—H⋯O hydrogen-bonding inter­action and an N—H⋯N inter­action generate ring motifs [graph sets *S*(6) and *S*(5), respectively]. In the crystal, mol­ecules form a chain through N—H⋯O hydrogen bonds, and these are extended by N—H⋯S hydrogen-bonding inter­actions into an infinite three-dimensional network. The crystal structure also exhibits weak C—H⋯π inter­actions.

## Related literature

For related structures, see: Qasem Ali *et al.* (2012 ▶, 2011*a* ▶,*b* ▶); Ali *et al.* (2012 ▶). For various biological activities of Schiff bases, see: Bhandari *et al.* (2008 ▶); Bhardwaj *et al.* (2010 ▶); Pandeya *et al.* (1999 ▶); Sridhar *et al.* (2002 ▶); Suryavanshi & Pai (2006 ▶). For cytotoxic and anti­cancer activities of isatin and its derivatives, see: Vine *et al.* (2009 ▶). For graph-set analysis, see Bernstein *et al.* (1995 ▶).
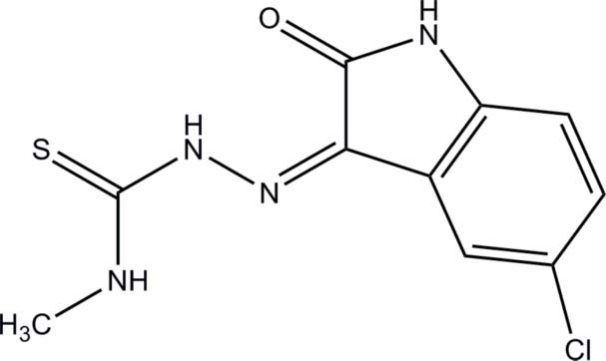


## Experimental

### 

#### Crystal data

C_10_H_9_ClN_4_OS
*M* *_r_* = 268.72
Orthorhombic, 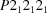

*a* = 6.2558 (1) Å
*b* = 10.1449 (1) Å
*c* = 18.5682 (2) Å
*V* = 1178.42 (3) Å^3^
*Z* = 4
Mo *K*α radiation
μ = 0.49 mm^−1^
*T* = 100 K
0.34 × 0.10 × 0.08 mm

#### Data collection

Bruker APEXII CCD diffractometer
Absorption correction: multi-scan (*SADABS*; Bruker, 2005 ▶) *T*_min_ = 0.853, *T*_max_ = 0.961
16807 measured reflections
4886 independent reflections
4072 reflections with *I* > 2σ(*I*)
*R*_int_ = 0.037

#### Refinement

*R*[*F* ^2^ > 2σ(*F* ^2^)] = 0.037
*wR*(*F* ^2^) = 0.078
*S* = 1.05
4886 reflections
167 parameters
H atoms treated by a mixture of independent and constrained refinement
Δρ_max_ = 0.41 e Å^−3^
Δρ_min_ = −0.32 e Å^−3^
Absolute structure: Flack (1983 ▶), 2074 Friedel pairs
Flack parameter: 0.01 (5)

### 

Data collection: *APEX2* (Bruker, 2005 ▶); cell refinement: *SAINT* (Bruker, 2005 ▶); data reduction: *SAINT*; program(s) used to solve structure: *SHELXS97* (Sheldrick, 2008 ▶); program(s) used to refine structure: *SHELXL97* (Sheldrick, 2008 ▶); molecular graphics: *SHELXTL* (Sheldrick, 2008 ▶); software used to prepare material for publication: *SHELXTL* and *PLATON* (Spek, 2009 ▶).

## Supplementary Material

Crystal structure: contains datablock(s) I, global. DOI: 10.1107/S1600536812007386/zs2177sup1.cif

Structure factors: contains datablock(s) I. DOI: 10.1107/S1600536812007386/zs2177Isup2.hkl

Supplementary material file. DOI: 10.1107/S1600536812007386/zs2177Isup3.cml

Additional supplementary materials:  crystallographic information; 3D view; checkCIF report

## Figures and Tables

**Table 1:** Hydrogen-bond geometry (Å, °) *Cg*2 is the centroid of the C1–C6 ring.

*D*—H⋯*A*	*D*—H	H⋯*A*	*D*⋯*A*	*D*—H⋯*A*
N4—H1N4⋯N2	0.88 (2)	2.27 (2)	2.6416 (18)	105.6 (15)
N4—H1N4⋯S1^i^	0.88 (2)	2.70 (2)	3.4972 (13)	152.2 (16)
N3—H1N3⋯O1	0.86 (2)	2.086 (19)	2.7526 (16)	134.3 (17)
N1—H1N1⋯O1^ii^	0.81 (2)	2.01 (2)	2.8161 (16)	175 (2)
C3—H3*A*⋯*Cg*2^iii^	0.95	2.59	3.38	141

Symmetry codes: (i) 
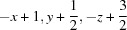
; (ii) 
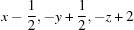
; (iii) 
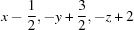
.

**Fig. 1.**
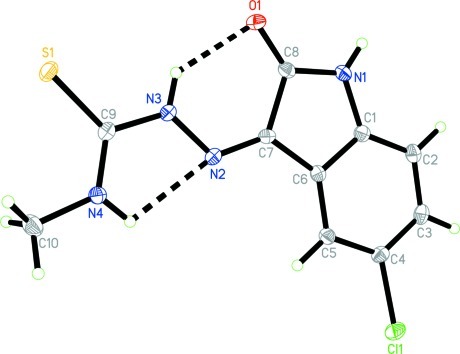
The molecular structure and the atom-numbering scheme of the title compound, with 50% probability displacement ellipsoids.

**Fig. 2.**
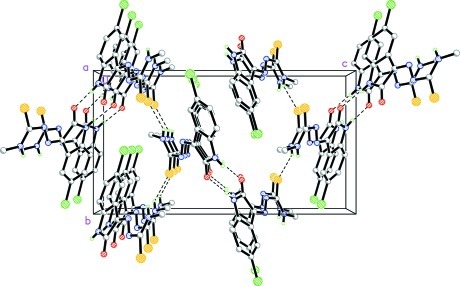
The crystal packing of the title compound viewed down the a axis. Hydrogen bonds are shown as dashed lines.

## supplementary crystallographic information

### Comment

Isatin (2,3-dioxindole) is an endogenous compound identified in humans, and its effect has been studied in a variety of systems. Biological properties of isatin and its derivatives include a range of actions in the brain, offer protection against bacterial (Suryavanshi & Pai, 2006) and fungal infections and possess anticonvulsant, anti-HIV (Pandeya *et al.*, 1999), anti-depressant and anti-inflammatory activities (Bhandari *et al.*, 2008). Recently, we reported the crystal structure of (*Z*)-2-(5-chloro-2-oxoindolin-3-ylidene)-*N*-methylhydrazinecarbothioamide (Qasem Ali *et al.*, 2012). In the present paper we describe the single-crystal X-ray diffraction study of title compound, C_10_H_9_ClN_4_OS (Fig. 1).

In this compound, the chain N2/N3/C9/S1/N4/C10 is connected to the nine-membered 5-chloroindolin-2-one ring system at C7. In this chain C7—N2—N3—C9 and C10—N4—C9—S1 torsion angles are -177.77 (13)° and 2.7 (2)°, respectively. The essentially planar conformation of the molecule is maintained by the cyclic intramolecular N3—H1N3···O1 hydrogen-bonding interaction together with the N4—H1N4···N2 interaction [graph sets *S*(6) and *S*(5), respectively (Bernstein *et al.*, 1995)] (Table 1).

In the crystal the molecules form chain substructures through intermolecular N1—H1N1···O1 hydrogen bonds and these are extended by N4—H1N4···S1 hydrogen-bonding interactions into an infinite a three-dimensional network (Table 1, Fig. 2). Weak C—H···π interactions are also present [C3—H···*Cg*2^iii^ = 3.38 Å], where *Cg*2 is the centroid of the C1—C6 ring. For symmetry code (iii), see Table 1.

### Experimental

The Schiff base has been synthesized by refluxing the reaction mixture of a hot ethanolic solution (30 ml) of 5-methyl-3-thiosemicarbazide (0.01 mol) and a hot ethanolic solution (30 ml) of 5-chloroisatin (0.01 mol) for 2 hr. The precipitate formed during reflux was filtered, washed with cold ethanol and recrystallized from hot ethanol. Yield (m.p.): 85% (568.4–569.0 K). The yellow crystals were grown in ethylacetate–DMF (3:1) by slow evaporation at room temperature.

### Refinement

Nitrogen bound H atoms were located in a difference Fourier map and were refined freely. The remaining H atoms were positioned geometrically and refined using a riding model with C—H = 0.95 Å (aromatic ring) and C—H = 0.98 Å (methyl group) with *U*_iso_(H) = 1.2*U*_eq_(aromatic C) and *U*_iso_(H) = 1.5*U*_eq_(methyl C). The highest residual electron density peak (0.41 eÅ^-3^) is located at 0.73 Å from Cl1 and the deepest hole (-0.32 eÅ^-3^) is located at 0.59 Å from Sl.

### Crystal data

**Table:**

C_10_H_9_ClN_4_OS	*D*_x_ = 1.515 Mg m^−^^3^
*M**_r_* = 268.72	Melting point = 568.4–569.0 K
Orthorhombic, *P*2_1_2_1_2_1_	Mo *K*α radiation, λ = 0.71073 Å
Hall symbol: P 2ac 2ab	Cell parameters from 5715 reflections
*a* = 6.2558 (1) Å	θ = 3.0–34.1°
*b* = 10.1449 (1) Å	µ = 0.49 mm^−^^1^
*c* = 18.5682 (2) Å	*T* = 100 K
*V* = 1178.42 (3) Å^3^	Needle, yellow
*Z* = 4	0.34 × 0.10 × 0.08 mm
*F*(000) = 552	

### Data collection

**Table:**

Bruker APEXII CCD diffractometer	4886 independent reflections
Radiation source: fine-focus sealed tube	4072 reflections with *I* > 2σ(*I*)
Graphite monochromator	*R*_int_ = 0.037
φ and ω scans	θ_max_ = 34.4°, θ_min_ = 2.2°
Absorption correction: multi-scan (*SADABS*; Bruker, 2005)	*h* = −8→9
*T*_min_ = 0.853, *T*_max_ = 0.961	*k* = −16→16
16807 measured reflections	*l* = −29→29

### Refinement

**Table:**

Refinement on *F*^2^	Secondary atom site location: difference Fourier map
Least-squares matrix: full	Hydrogen site location: inferred from neighbouring sites
*R*[*F*^2^ > 2σ(*F*^2^)] = 0.037	H atoms treated by a mixture of independent and constrained refinement
*wR*(*F*^2^) = 0.078	*w* = 1/[σ^2^(*F*_o_^2^) + (0.0318*P*)^2^ + 0.1624*P*] where *P* = (*F*_o_^2^ + 2*F*_c_^2^)/3
*S* = 1.05	(Δ/σ)_max_ = 0.001
4886 reflections	Δρ_max_ = 0.41 e Å^−^^3^
167 parameters	Δρ_min_ = −0.32 e Å^−^^3^
0 restraints	Absolute structure: Flack (1983), 2074 Friedel pairs
Primary atom site location: structure-invariant direct methods	Flack parameter: 0.01 (5)

### Special details

**Table:**

Geometry. All e.s.d.'s (except the e.s.d. in the dihedral angle between two l.s. planes) are estimated using the full covariance matrix. The cell e.s.d.'s are taken into account individually in the estimation of e.s.d.'s in distances, angles and torsion angles; correlations between e.s.d.'s in cell parameters are only used when they are defined by crystal symmetry. An approximate (isotropic) treatment of cell e.s.d.'s is used for estimating e.s.d.'s involving l.s. planes.
Refinement. Refinement of *F*^2^ against ALL reflections. The weighted *R*-factor *wR* and goodness of fit *S* are based on *F*^2^, conventional *R*-factors *R* are based on *F*, with *F* set to zero for negative *F*^2^. The threshold expression of *F*^2^ > σ(*F*^2^) is used only for calculating *R*-factors(gt) *etc*. and is not relevant to the choice of reflections for refinement. *R*-factors based on *F*^2^ are statistically about twice as large as those based on *F*, and *R*- factors based on ALL data will be even larger.

### Fractional atomic coordinates and isotropic or equivalent isotropic displacement parameters (Å^2^)

**Table:**

	*x*	*y*	*z*	*U*_iso_*/*U*_eq_	
Cl1	−0.30754 (7)	0.94253 (3)	0.877263 (19)	0.02071 (9)	
S1	0.74171 (6)	0.27814 (3)	0.785567 (19)	0.01723 (8)	
O1	0.15801 (18)	0.26544 (10)	0.93453 (5)	0.0176 (2)	
N1	−0.1313 (2)	0.39745 (12)	0.96526 (6)	0.0154 (3)	
N2	0.2697 (2)	0.50267 (11)	0.84352 (6)	0.0131 (2)	
N3	0.4038 (2)	0.39983 (12)	0.83590 (6)	0.0144 (2)	
N4	0.6135 (2)	0.52656 (12)	0.76196 (7)	0.0147 (2)	
C1	−0.2019 (3)	0.52525 (13)	0.94808 (7)	0.0138 (3)	
C2	−0.3841 (3)	0.58915 (15)	0.97154 (7)	0.0160 (3)	
H2A	−0.4830	0.5474	1.0029	0.019*	
C3	−0.4167 (2)	0.71814 (15)	0.94706 (7)	0.0163 (3)	
H3A	−0.5411	0.7650	0.9613	0.020*	
C4	−0.2680 (3)	0.77827 (13)	0.90194 (7)	0.0148 (3)	
C5	−0.0871 (2)	0.71336 (14)	0.87730 (7)	0.0139 (3)	
H5A	0.0114	0.7551	0.8458	0.017*	
C6	−0.0562 (2)	0.58458 (14)	0.90075 (7)	0.0124 (3)	
C7	0.1086 (2)	0.48782 (13)	0.88630 (7)	0.0127 (3)	
C8	0.0532 (3)	0.36840 (14)	0.93058 (7)	0.0139 (3)	
C9	0.5824 (2)	0.41081 (13)	0.79306 (7)	0.0127 (3)	
C10	0.7903 (3)	0.55324 (15)	0.71346 (8)	0.0201 (3)	
H10A	0.8041	0.6486	0.7065	0.030*	
H10B	0.7634	0.5107	0.6670	0.030*	
H10C	0.9228	0.5184	0.7342	0.030*	
H1N4	0.516 (4)	0.588 (2)	0.7671 (10)	0.033 (6)*	
H1N3	0.383 (3)	0.328 (2)	0.8590 (10)	0.032 (6)*	
H1N1	−0.189 (4)	0.3462 (19)	0.9928 (11)	0.035 (6)*	

### Atomic displacement parameters (Å^2^)

**Table:**

	*U*^11^	*U*^22^	*U*^33^	*U*^12^	*U*^13^	*U*^23^
Cl1	0.0223 (2)	0.01646 (14)	0.02338 (15)	0.00725 (15)	−0.00058 (16)	0.00258 (13)
S1	0.01584 (18)	0.01392 (14)	0.02193 (15)	0.00320 (15)	0.00197 (16)	−0.00174 (13)
O1	0.0214 (6)	0.0140 (4)	0.0174 (4)	0.0042 (5)	−0.0002 (4)	0.0032 (4)
N1	0.0183 (7)	0.0138 (5)	0.0141 (5)	−0.0012 (5)	0.0033 (5)	0.0017 (4)
N2	0.0134 (6)	0.0118 (4)	0.0140 (5)	0.0016 (5)	−0.0007 (5)	−0.0013 (4)
N3	0.0150 (7)	0.0122 (5)	0.0161 (5)	0.0026 (5)	0.0026 (5)	0.0008 (4)
N4	0.0129 (6)	0.0138 (5)	0.0175 (5)	0.0005 (5)	0.0018 (5)	0.0017 (4)
C1	0.0155 (7)	0.0135 (5)	0.0123 (5)	−0.0014 (5)	−0.0011 (6)	−0.0011 (4)
C2	0.0145 (7)	0.0189 (6)	0.0148 (6)	−0.0014 (6)	0.0018 (6)	−0.0014 (5)
C3	0.0130 (7)	0.0201 (6)	0.0159 (6)	0.0021 (6)	−0.0002 (6)	−0.0040 (5)
C4	0.0157 (7)	0.0142 (5)	0.0146 (5)	0.0022 (6)	−0.0034 (6)	−0.0002 (5)
C5	0.0144 (7)	0.0146 (5)	0.0128 (5)	0.0000 (6)	0.0003 (6)	0.0013 (5)
C6	0.0119 (7)	0.0145 (6)	0.0108 (5)	−0.0006 (5)	−0.0009 (5)	−0.0003 (4)
C7	0.0142 (7)	0.0109 (5)	0.0129 (6)	0.0000 (5)	−0.0013 (5)	0.0010 (4)
C8	0.0168 (7)	0.0135 (6)	0.0113 (5)	−0.0019 (6)	−0.0007 (6)	0.0010 (4)
C9	0.0115 (7)	0.0129 (5)	0.0136 (6)	0.0000 (5)	−0.0023 (5)	−0.0026 (5)
C10	0.0162 (8)	0.0229 (7)	0.0211 (6)	−0.0025 (6)	0.0021 (6)	0.0042 (6)

### Geometric parameters (Å, º)

**Table:**

Cl1—C4	1.7458 (14)	C1—C6	1.402 (2)
S1—C9	1.6806 (14)	C2—C3	1.400 (2)
O1—C8	1.2354 (18)	C2—H2A	0.9500
N1—C8	1.355 (2)	C3—C4	1.392 (2)
N1—C1	1.4062 (18)	C3—H3A	0.9500
N1—H1N1	0.81 (2)	C4—C5	1.387 (2)
N2—C7	1.292 (2)	C5—C6	1.3906 (19)
N2—N3	1.3463 (17)	C5—H5A	0.9500
N3—C9	1.376 (2)	C6—C7	1.449 (2)
N3—H1N3	0.86 (2)	C7—C8	1.5044 (19)
N4—C9	1.3229 (18)	C10—H10A	0.9800
N4—C10	1.4520 (19)	C10—H10B	0.9800
N4—H1N4	0.88 (2)	C10—H10C	0.9800
C1—C2	1.382 (2)		
			
C8—N1—C1	111.11 (12)	C4—C5—C6	117.14 (13)
C8—N1—H1N1	122.4 (16)	C4—C5—H5A	121.4
C1—N1—H1N1	126.4 (16)	C6—C5—H5A	121.4
C7—N2—N3	117.41 (11)	C5—C6—C1	120.62 (14)
N2—N3—C9	120.27 (12)	C5—C6—C7	132.66 (13)
N2—N3—H1N3	121.0 (14)	C1—C6—C7	106.73 (12)
C9—N3—H1N3	118.7 (14)	N2—C7—C6	126.15 (12)
C9—N4—C10	123.25 (13)	N2—C7—C8	127.55 (13)
C9—N4—H1N4	119.0 (14)	C6—C7—C8	106.30 (12)
C10—N4—H1N4	117.5 (14)	O1—C8—N1	127.44 (13)
C2—C1—C6	122.18 (13)	O1—C8—C7	126.25 (14)
C2—C1—N1	128.31 (14)	N1—C8—C7	106.31 (12)
C6—C1—N1	109.52 (13)	N4—C9—N3	116.33 (13)
C1—C2—C3	117.14 (14)	N4—C9—S1	125.98 (12)
C1—C2—H2A	121.4	N3—C9—S1	117.69 (10)
C3—C2—H2A	121.4	N4—C10—H10A	109.5
C4—C3—C2	120.50 (14)	N4—C10—H10B	109.5
C4—C3—H3A	119.7	H10A—C10—H10B	109.5
C2—C3—H3A	119.7	N4—C10—H10C	109.5
C5—C4—C3	122.37 (13)	H10A—C10—H10C	109.5
C5—C4—Cl1	118.82 (11)	H10B—C10—H10C	109.5
C3—C4—Cl1	118.79 (12)		
			
C7—N2—N3—C9	−177.77 (13)	N3—N2—C7—C6	−178.53 (13)
C8—N1—C1—C2	178.80 (14)	N3—N2—C7—C8	1.2 (2)
C8—N1—C1—C6	−0.92 (16)	C5—C6—C7—N2	−2.2 (3)
C6—C1—C2—C3	−1.2 (2)	C1—C6—C7—N2	177.97 (14)
N1—C1—C2—C3	179.15 (13)	C5—C6—C7—C8	177.99 (15)
C1—C2—C3—C4	−1.0 (2)	C1—C6—C7—C8	−1.83 (15)
C2—C3—C4—C5	2.3 (2)	C1—N1—C8—O1	179.09 (15)
C2—C3—C4—Cl1	−176.16 (11)	C1—N1—C8—C7	−0.27 (16)
C3—C4—C5—C6	−1.3 (2)	N2—C7—C8—O1	2.1 (3)
Cl1—C4—C5—C6	177.14 (11)	C6—C7—C8—O1	−178.07 (14)
C4—C5—C6—C1	−0.9 (2)	N2—C7—C8—N1	−178.50 (14)
C4—C5—C6—C7	179.33 (14)	C6—C7—C8—N1	1.30 (15)
C2—C1—C6—C5	2.1 (2)	C10—N4—C9—N3	−178.06 (13)
N1—C1—C6—C5	−178.12 (13)	C10—N4—C9—S1	2.7 (2)
C2—C1—C6—C7	−178.02 (13)	N2—N3—C9—N4	1.08 (19)
N1—C1—C6—C7	1.72 (15)	N2—N3—C9—S1	−179.58 (10)

### Hydrogen-bond geometry (Å, º)

*Cg*2 is the centroid of the C1–C6 ring.

**Table:**

*D*—H···*A*	*D*—H	H···*A*	*D*···*A*	*D*—H···*A*
N4—H1*N*4···N2	0.88 (2)	2.27 (2)	2.6416 (18)	105.6 (15)
N4—H1*N*4···S1^i^	0.88 (2)	2.70 (2)	3.4972 (13)	152.2 (16)
N3—H1*N*3···O1	0.86 (2)	2.086 (19)	2.7526 (16)	134.3 (17)
N1—H1*N*1···O1^ii^	0.81 (2)	2.01 (2)	2.8161 (16)	175 (2)
C3—H3*A*···*Cg*2^iii^	0.95	2.59	3.38	141

Symmetry codes: (i) −*x*+1, *y*+1/2, −*z*+3/2; (ii) *x*−1/2, −*y*+1/2, −*z*+2; (iii) *x*−1/2, −*y*+3/2, −*z*+2.
